# Developing culturally-responsive health promotion: insights from cultural experts

**DOI:** 10.1093/heapro/daad022

**Published:** 2023-04-17

**Authors:** Mele A Look, Gregory G Maskarinec, Māpuana de Silva, Kamuela Werner, Tricia Mabellos, Donna-Marie Palakiko, Stacy L Haumea, Joseph Gonsalves, Aukahi A Seabury, Jetney Kahaulahilahi Vegas, Cappy Solatorio, Joseph Keaweʻaimoku Kaholokula

**Affiliations:** Department of Native Hawaiian Health in Honolulu, University of Hawaiʻi, John A. Burns School of Medicine, Honolulu, HI, USA; Hālau Mōhala ʻIlima, Kāohao, HI, USA; University of HawaiʻI, John A. Burns School of Medicine, Honolulu, HI, USA; Hālau Mōhala ʻIlima, Kāohao, HI, USA; Department of Native Hawaiian Health, University of Hawaiʻi, John A. Burns School of Medicine, Honolulu, HI, USA; Department of Native Hawaiian Health, University of Hawaiʻi, John A. Burns School of Medicine, Honolulu, HI, USA; University of Hawaiʻi, School of Nursing, Ke Ola Mamo, Native Hawaiian Health Care System, Honolulu, HI, USA; Mauli Ola, Hilo, HI, USA; Hui No ke Ola Pono, Native Hawaiian Health Care System, Waikuku, HI, USA; I Ola Lāhui, Honolulu, HI, USA; Waiʻanae Coast Comprehensive Health Center, Waianae, HI, USA; Kula no nā Poʻe Hawaiʻi, Honolulu, HI, USA; Department of Native Hawaiian Health, University of Hawaiʻi, John A. Burns School of Medicine, Honolulu, HI, USA

**Keywords:** dance, indigenous, Hawaiian, community health promotion, cardiovascular disease

## Abstract

Culturally-responsive health promotion initiatives are important to the creation of health equity for Indigenous and minority populations and these initiatives are complex and time-intensive to establish. The knowledge and resources of cultural experts are often pivotal in programs, yet there is minimal research on effective collaborations. The KāHOLO Project demonstrated strong success in the management of uncontrolled hypertension in the high-risk Indigenous population through a 6-month program based on the Hawaiian cultural dance of hula. This program was developed utilizing a community-based participatory research approach and implemented by cultural experts. To better understand the effectiveness of the research endeavor and program, six experienced hula experts and educators who delivered the community-based program were interviewed. As skilled and trusted cultural experts they set a safe supportive learning environment that promoted health and cultural goals. They articulated it was important that the program maintained cultural priorities and integrity. Through the methodical establishment of mutual respect, cooperation on research protocols and requirements was achieved. The development of cultural experts as health allies offers important inroads to the inclusion of minority and Indigenous cultures in health programming.

## INTRODUCTION

The need for culturally-responsive health promotion initiatives for Indigenous and minority populations is well established (Kaholokula *et al*., 2018; Harding and Oetzel, 2019; Walters *et al*., 2020). It is also known that developing and implementing such initiatives are complex and difficult (Nápoles and Stewart, 2018; Soong *et al*., 2021). The challenges include the necessity of authentic community engagement practices (DHHS, 2011; O’Mara-Eves *et al*., 2015) and systemic barriers in our present biomedical-driven health system (Golden and Wendel, 2020). From the conception to the implementation of culturally-responsive health promotion programs, the engagement of cultural experts can be instrumental (Harding and Oetzel, 2019). Yet, research is lacking on the best practices for collaborating with cultural experts for these activities and how to incorporate the insights they bring.

For the past decade, the Department of Native Hawaiian Health, at the University of Hawaiʻi’s medical school has worked in partnership with Native Hawaiian community leaders and cultural experts to formulate cardiovascular health promotion initiatives based on the indigenous Hawaiian cultural dance of hula. Most recently, the Departmentʻs KāHOLO Project utilized a community-based participatory research (CBPR) approach to develop and evaluate, through a randomized wait-list control trial (RCT), a culturally grounded hypertension (HTN) management program named *Ola Hou i ka Hula* (restore health through hula), in Native Hawaiians with high risk for HTN (Kaholokula *et al*., 2017a, b). The RCT found the Ola Hou intervention group, had a significantly greater reduction in systolic and diastolic blood pressure than the wait-list control group. In addition, 43% of Ola Hou group participants improved from Stage 3 HTN (systolic blood pressure > 140 mmHg) to Stage 2 HTN (systolic blood pressure > 130 mmHg) compared with 21% of the control group. Importantly, the Ola Hou group maintained blood pressure improvements at the 12-month testing assessment, which was 6-months after the conclusion of the Ola Hou program activities. Lastly, the 10-year cardiovascular disease risk reduction for the Ola Hou group was two times greater than the control group. The KāHOLO Project concluded that the Ola Hou program was effective at leveraging an Indigenous cultural dance practice for health promotion of HTN management and cardiovascular health in a high-risk population (Kaholokula *et al*., 2021).

Our purpose in adding a qualitative study, which is reported here, to the KāHOLO Project was to gain an in-depth understanding of how the cultural experts, who were key to delivering the intervention program, contributed to achieving the health objectives. We also sought to learn what these experts had to say about the program, and gather their thoughts regarding being part of a health initiative and a research effort. Responses gathered may be of use to the development and implementation of culturally-responsive interventions as a modality to improve health for minority and Indigenous communities.

### Hula and cultural expertise

The cultural dance of hula is an iconic symbol of Hawai‘i whose ancient origins included both sacred and secular purpose, and in contemporary times often function as a form of creative expression and cultural identity, as well as, its traditional purpose of preserving history and stories (Stillman, 1998; Kaeppler, 2004). Hula is an inclusive cultural practice, that although has strong appeal to Native Hawaiians, welcomes all genders, and all ethnicities. Prior research established that the health benefits of hula included physical, social, and spiritual aspects (Look *et al*., 2014; Usagawa *et al*., 2014; Kaholokula *et al*., 2017a).

Traditionally, hula is exclusively taught by a *kumu hula* (hula expert, educator) or their designated *alakaʻi hula* (hula leader), typically a lead student. Kumu hula are community leaders and are widely sought after for their cultural knowledge and skills. Hula dances are taught through learning traditional movement motifs for the upper and lower body that function rhythmically to keep the dancer in time and to keep groups of dancers coordinated. Stylized hand and arm gestures interpret aspects of the poetic text of the accompanying *mele* (song or chant) (Stillman, 2007). Superior hula training undertaken by kumu and ʻalakaʻi, takes years if not decades, and requires edification in the Hawaiian language, customs, cultural protocol, history, and metaphoric and poetic insight into the performance chants and songs (Kaeppler, 1976). In Hawaiʻi, hula remains popular with 203 *hālau hula* (traditional hula and cultural arts training centers) across the eight main islands and commonplace formal performances, not just in tourist venues, but more so in theatre, concerts, and school productions, as well as informal presentations at social and family functions.

### KāHOLO Project research protocol

The KāHOLO Projectʻs 6-month Ola Hou program was conducted at eight urban and rural community sites by a community facilitator who provided health education and logistical support, and a kumu hula or alakaʻi hula who served as the dance educator. The dance educators were responsible for leading the 60-min hula classes twice a week for the first 3 months, and once a month for the remaining three months of the program. Before classes began, all hula educators received 6 hr of orientation and training from research staff and a highly respected hula expert, who served as a cultural consultant to the study. The training provided an overview of the research and health promotion objectives, and set forth guidelines to establish consistency across sites (Kaholokula, 2017b).

The hula educators also participated in fidelity checks which were conducted by the study’s cultural consultant and consisted of observation of a class and evaluation of predetermined program criteria. If inconsistency in program delivery was observed, research staff was informed and feedback was provided by a lead KāHOLO investigator to the hula educator and the respective site facilitator. There was a total of 10 trained hula educators involved in the KāHOLO Project.

## METHODS

All 10 hula educators were invited to participate in the interviews. Because of consistency across responses, it was concluded that saturation was achieved when six educators were interviewed. The six respondents participated individually in face-to-face, open-ended, semi-structured 30–90 min conversations (key informant interviews) that were recorded, with the consent of the respondent. The interviews were held at a time and location for the convenience of respondents, often they were conducted at the organization where the Ola Hou program classes were held. The interviewers were not directly involved with the KāHOLO study to provide a neutral environment for commentary from the respondents. Demographic information of the respondents was collected from their interviews and from a registration sheet they completed upon joining the KāHOLO Project. This qualitative study was reviewed by the University of Hawai’i’s institutional review board and approved as exempt.

The respondents interviewed included: five females and one male, with an age ranging from 27 to 67 years. All respondents formally taught hula for at least 5 years, with two having taught hula for more than 10 years. In addition to experience teaching in private classes at a hālau hula, the respondents reported providing dance instruction for special events such as school or church hula performances, as well as, to family and friends. Four of the respondents self-identified as a kumu hula and two as ‘alaka‘i hula. Four of the respondents self-identified as Native Hawaiian. Five of the six community-based partnering organizations were represented in the interviews. These organizations were located across three Hawaiian Islands, four were in urban and two in rural areas (Table 1). Most of the respondents self-reported having at least one cardiovascular disease risk factor.

**Table 1:** Hula educators demographics

Demographic	*n*	%
Gender		
Female	5	83.3
Male	1	16.7
Age		
18–29 years	1	16.7
30–59 years	3	50.0
60 + years	2	33.3
Ethnicity		
Native Hawaiian	4	66.7
Non-Hawaiian	2	33.3
Years teaching hula		
0–4 years	0	0.0
5–9 years	2	33.3
10+ years	4	66.7
Hula position/title		
Kumu	4	66.7
‘Alaka‘i	2	33.3
Residence		
Urban	4	66.7
Rural	2	33.3

Following a semi-structured interview outline, the respondents were encouraged to discuss challenges, unexpected issues, and strategies used in teaching the Ola Hou hula classes. Did they now connect hula and health in new ways? What benefits, challenges, or risks, such as potential threats to the cultural integrity of the indigenous dance traditions, did they experience by being involved in a biomedical research study involving hula? Did their training for the study, or teaching the Ola Hou program, influence them to alter the ways they taught hula in their private classes? We also asked these educators to review and explain: (i) aspects of Hawaiian culture added as part of their classes, (ii) mele and dance selection, (iii) whether becoming involved with the Ola Hou program changed their attitudes toward their health, and (iv) whether they had any suggestions for how a similar program could be implemented and sustained when the project ends.

### Data analysis

Interviews were transcribed by a lead investigator. While all interviews were conducted in English, most respondents utilized words and phrases in the Hawaiian language during the interviews. After the initial transcription, all Hawaiian words and phrases were translated by a bilingual research assistant. All translations were then reviewed by a bilingual member of the KāHOLO Project to ensure accuracy.

The interviews were evaluated by thematic analysis following standard qualitative methods (Silverman, 2013). First, four members of the research team (three with prior hula training), independently reviewed all transcripts and established general domains suggested by the responses of the participants to the questions. Then, team members again independently reviewed the transcripts to identify repeating themes within each domain, noting other themes that emerged with high frequency. Next, a fifth investigator with Hawaiian culture, language, and hula expertise reviewed the transcripts to determine if additional themes or domains could be identified or clarified. Finally, the frequency of various themes was established through the coding of all transcripts (Dey, 2003). While some of those interviewed expressed their views more succinctly than others, the quotes in the results that follow are drawn from all but one respondent. Respondents were allocated a number (1–6) to ensure their anonymity.

## FINDINGS

Our findings highlight the strong support by the hula educators for the utilization of the cultural practice of hula for health promotion. The most frequently mentioned reason respondents gave for their decision to join the KāHOLO research effort was, ‘*to help my community improve their health*’. Throughout the interviews, respondents: endorsed hula as a means for health promotion, identified key aspects of their teaching approach, elaborated on specific ways they integrated cultural lessons, and described how they contributed to the health promotion program goals and participant attendance. There were six key points on which all hula educators agreed:

1) **Participant commitment is essential**. The hula educators understood the Ola Hou classes were designed to promote self-efficacy and self-confidence in both hula and HTN self-management; therefore, participants must be committed to self-improvement. As Respondent 2 noted:

*[T]he purpose of the study is to push them; to see what they’re capable of, to build their capacity: their own physical capacity, cultural capacity, mental capacity. And so you can say, I know you can do it.*

*This respondent elaborated, ‘if the heart is willing, if the na’au [seat of intellect and emotions] is willing, anything can happen. If they’ve made the first step by coming, that’s all you really need. And if they enjoy themselves, that breaks down so many walls’.*

Another educator, Respondent 3, further developed on this point, described how the building of personal relationships within the class enhanced the dancers commitment: *‘The value of the relationships; they share a challenge in their life—all are looking for something’.* The importance of the relationships between dancers, as well as the relationship between the hula educators and the dancers, were frequently mentioned by the respondents.

Respondent 6 describes the openness that was established in the classes, and her affinity with the dancers. ‘*The participants are so kind, they are so gentle, they have their backgrounds, their histories, and their stories. But they come, they share openly about their health issues so that I think is healthy in itself. To not be ashamed of what’s going on’.*

2) **Both research and hula cultural protocols are necessary and appropriate.** The hula educators appreciated the Ola Hou training they received and expressed how it reinforced their teaching approach. The training emphasized the classes be consistent with the traditional hula training curriculum that includes lessons in Hawaiian culture, language, and history as it related to the subject matter of the dances. Ola Hou classes were also structured to increase extended periods of physical exertion through continuous dancing. Respondent 1: ‘*I really enjoyed teaching it [hula] the way it was taught to us [in the hula educators training]’.* It was also noted by Respondent 5 that participants responded well to the health focus and experiential teaching approach. *‘…they enjoy the presentations about their medications, the medical side of the program—the incorporation of healthy eating and nutrition, so they find out that it has nothing to do with wanting them—like forcing them to lose weight, change their eating habits, it’s just bringing awareness and it’s all through demonstration’.*
3) **Song selection is of crucial importance.** The hula educators had the prerogative to select the dances taught in their classes. During their training for the study, the educators were provided with examples of hula dances with appropriate complexity and physical exertion for the Ola Hou program participants, and although the lyrics were entirely in the Hawaiian language they included clear metaphorical imagery, as well as, Hawaiian words familiar to most people who live in Hawai‘i. One example dance was ‘Kawika’, composed in the 19th Century as a tribute to King David Kalakaua who brought back hula to public performances after it was banned under pressure from foreign missionaries and their descendants (Barrere, 1980). The another example dance was a contemporary hula about a well-known musical family and their beloved seaside home. All the educators were very familiar with both dances and some included these dances in their Ola Hou class repertoire. Several educators noted they chose to include hula relevant to the geographic location where the participants lived. Respondent 2 explained, that place-based hula establishes a relationship between the dancer and deep meanings and feelings attached to Hawaiian places. She also observed that songs provide *‘a structure to follow, it worked, using hula, weaving in those cultural metaphors, those layers of meaning, so as to hook them into something, to have them wanting to continue…’* Another, Respondent 5, expanded on this, she specifically selected a hula, ‘Hōlei’, whose lyrics are from classic Hawaiian literature set in the general location of that siteʻs Ola Hou classes. *‘…the first hula I taught them was a mele of Puna [on Hawaiʻi Island]. A mele that talks about [goddess] Hiʻiakaikapoliopele’s journey’.* By utilizing a chant from this epic tale she was able to share with participants: a quintessential Hawaiian viewpoint of the relationship to the natural world, as well as utilize the metaphor of the main character, the goddess Hiʻiaka’s long and arduous journey to their journey to improving their health. *‘every motion I teach them, I explain to them—it’s not just a motion for hula, it’s a motion for health, healthy living’.* After many weeks of learning the dance, she culminated the experience by taking the dancers to the specific location the song talks about *‘…the dancers danced right there looking at Hōlei and that was really special’.* Methodically, the hula educators set a curriculum that built hula skills and competency and tied it to health promotion and a foundation of self-efficacy.
4) **Maintaining cultural integrity and Hawaiian values were achieved.** None of the educators worried about losing the cultural integrity of hula because it was taught for a health objective and as part of a research project, and all expressed that they were able to incorporate traditional values into the classes. Respondent 3: *‘If it is [just] to teach the choreography, that’s different, that is about explaining the motions. [But by] making the[song] meaning personal—so they can connect the motion emotionally to the song, for me that’s is hula. It’s not just memorizing where my hand goes, it’s—why is that the motion for that word?’* The educators articulated that hula choreography has the specific purpose of storytelling. They understood and expressed how hula connects specific gestures to polysemic images of history, society, and the cosmos within a uniquely Hawaiian perspective, and they agreed this perspective need not be compromised when it is used in a research project or a health program.
5) **The program should be sustained and disseminated further**. The educators expressed significant support for the Ola Hou program and advocated having the Ola Hou program established across communities. They believed the program would be popular because of the popularity and accessibility of hula. Learning hula gave those interested in improving their health, an opportunity to learn Hawaiian culture. ‘*I think we are creating the pathway, not just for the future, but also for our kūpuna [elders and ancestors] who are still with us*’, Respondent 2 concluded. Another, Respondent 1, commented: ‘*The program is awesome... Life is ever learning*’.
6) **The cultural practice of hula is intrinsically related to health.** The educators found that being part of a culturally-grounded research study increased their insight into how the cultural practice of hula was intrinsically related to health, and how this insight also impacted their own health. Respondent 3 shared ‘*teaching [the Ola Hou program] inspires me to do more, to take control of my health more’.* The hula educators expressed there could be a multitude of benefits, including an array of health benefits if Hawaiians of all ages had an opportunity to learn this cultural practice. And they acknowledged there are personal, political, economic, and cultural issues that may prevent this from happening. As Respondent 1, expressed, *‘The relation of culture and hula, to health is more explicit now’.*, Respondent 6, pointed out that the benefits are comprehensive for mind, body, and spirit. ‘*I’m more aware of breathing, take a breath and stretch, that kind of thing, just being mindful of the health of everybody, all the haumana [students] so that’s been a really big benefit...hula is pule [prayer]’.*

## DISCUSSION

The findings indicate that the hula educators served a key role in establishing the cultural relevancy of health promotion in the Ola Hou program. The hula educators, with their significant cultural training, understood that relational connections between people and places are a fundamental Hawaiian value and critical to engagement in learning (Native Hawaiian Education Council, 2002; Kamaka, 2017; Matenga-Ikihele *et al*., 2021). They, through their expertise, established a safe supportive environment that encouraged self-confidence and efficacy in learning new skills, information, and behaviors that applied to both hula and health promotion. As experienced educators, they understood the role of participant commitment and promoted this commitment through various teaching approaches.

### Establishing cultural-relevancy

Cultural relevancy, particularly for Indigenous populations, is widely understood as essential in developing meaningful and relevant initiatives (Kanaiapuni *et al*., 2017; Kaholokula *et al*., 2018; Walters *et al*., 2020; Matenga-Ikihele *et al*., 2021). Respondents highlighted ways they facilitated the cultural connections through experiential teaching, utilization of Hawaiian values, and highlighting local histories and knowledge. To objectively understand how the hula educators incorporated cultural relevancy, we reviewed established guidelines for creating Hawaiian culturally healthy and responsive learning environments as they related to the hula training activities of the Ola Hou program. These guidelines were developed through a collaboration between the US federally-established Native Hawaiian Education Council and the University of Hawai‘i (Native Hawaiian Education Council, 2002) with the objective to “nurture the culturally healthy and responsive citizens” and present effective methodologies to develop responsive educators, curricula, schools, and other places of learning. The review found that in each of the 16 guidelines, the hula educators used various strategies and activities to establish cultural relevancy. Table 2 shows the application of six guidelines, providing descriptions, guideline examples, and how hula educators applied activities that fulfilled the guidelines’ objectives. A complete description of all 16 guidelines can be found in the online Supplementary Material (Native Hawaiian Education Council, 2002).

**Table 2:** Native Hawaiian Education Guidelines and Ola Hou Application

Guideline description	Guideline example	Ola hou hula educators application of guidelines
Incorporate cultural traditions, language, history, and values in meaningful holistic processes to nourish the emotional, physical, mental/intellectual, social, and spiritual well-being of the learning community that promote healthy *mauli* (life force) and *mana* (power, authority, privilege)	Model culturally appropriate behaviour in teaching	Establishing safe, familial learning environment and relationships between participants and between hula educator and participants
Maintain practices that perpetuate Hawaiian heritage, traditions, and language to nurture one’s mauli and perpetuate the success of the whole learning community	Provide opportunities to learn through observation and hands-on demonstration of cultural knowledge and skills	Learning of hula dances as means to increase cultural knowledge of Hawaiian language, history, and practices
Sustain respect for the integrity of one’s own cultural knowledge and provide meaningful opportunities to make new connections among other knowledge systems	Provide experiences that encourage learners to appreciate the uniqueness of other cultures	Sharing of heritage and various multi-cultural food And experience at ceremonial events
Instil a desire for lifelong exploration of learning, teaching, leading, and reflecting to pursue standards of quality and excellence	Demonstrate quality and excellence through product and performance	Inclusion of a traditional presentation of knowledge of knowledge or skills, which included presentation of learned hula repertoire
Provide safe and supportive places to nurture the physical, mental/intellectual, social, emotional, and spiritual health of the total community	Utilize multiple instructional strategies and apply those strategies appropriately and flexibly in response to the instructional environment in which they are situated (i.e. singing, learning to speak Hawaiian)	Use of songs in the Hawaiian language and including singing of Hawaiian language songs in the warm-up walking-singing portion of classes
Engage in activities independently or collaboratively with community members to perpetuate traditional ways of knowing, learning, teaching, and leading to sustain cultural knowledge and resources within the learning community	Provide opportunities for students to learn through observation and hands-on demonstrations of cultural knowledge and skills	Teaching place-based hula and including outings to historic areas
Develop an understanding of Hawaiian language, history, culture, and values through an indigenous perspective to foster a sense of self, place, community and global connection	Use local expertise, especially knowledgeable *kūpuna* (elders) as resource teachers/resources in classrooms and on excursions	Use of excursions to learn about songs used in dances. Use the knowledge of the participants to enrich discussions

Source: Native Hawaiian Education Council (2002).

### Building Collaborations with Cultural Experts

The importance of cultural understanding when delivering health promotion interventions to Indigenous and minority populations is well established (Barker *et al*. 2017; Came *et al*., 2019; Walters *et al*., 2020). Community health workers and peer counselors with ethnic-specific cultural background, have continually shown to have insights, and knowledge beneficial to health program development and delivery (Pinto *et al*., 2014; Suina, 2016). There is less understanding on the sucessful engagement of cultural experts who have not been involved in health promotion efforts. These individuals hold specialized expertise, knowledge, and skills in defined cultural areas such as traditional subsistence, language, and the creative arts.

The Ola Hou dance educators confirmed that the cultural integrity of the hula traditions was maintained in the Ola Hou health intervention program and credited this to the research study’s orientation training, and alignment with traditional hula teaching principles. They also noted that the autonomy they were provided, such as in dance selection, allowed them to create interest and craft lessons around local historic sites, stories, and Hawaiian cultural values. Notably, none of the educators objected to the fidelity checks or guidelines of hula dance instruction provided during the orientation training. The acceptance of research and health program protocols by the hula experts demonstrates that collaboration can be achieved particularly when mutual respect and trust are established.

To create trust, KāHOLO researchers acknowledged the skills and experience of the hula educators by providing latitude in the delivery of their expertise. Specifically, the research protocol supported traditional hula training approaches, and provided flexibility in areas important to the cultural practice such as song selection and choreography. It is likely establishing trust facilitated the acceptance of sensitive research requirements, such as fidelity checks. It was also meaningful that several members of the research leadership team were cultural practitioners of hula. Embedding cultural knowledge within the research team was continually helpful for communication and understanding the nuances of relationships and expectations. An important aspect of this achievement was by ensuring that a majority of the research team and our external scientific advisory group were cultural practitioners of hula, and that some members had fluency in the Hawaiian language. (Look *et al*., 2012). Also, beginning with our initial studies more than a decade ago, we conducted ongoing formal and informal consultation with widely respected kumu hula throughout the research efforts (Look *et al*., 2014; Kaholokula *et al*., 2017b). This approach follows the Hawaiian cultural protocol of consultation and acknowledgment of *‘ike kūpuna* (past wisdom). We believe, not only did we gain valuable guidance through process but also received respect by the larger community of hula practitioners by demonstrating our commitment to traditional Native Hawaiian values and practices.

The Ola Hou program provides an example of how cultural traditions, such as dance, can be used as a foundation for health promotion initiatives in Indigenous populations. Cultural practitioners, such as the hula educators, have a deep comprehension of their communities and bring skills and trust to achieving goals, be it dance or health. The collaboration empowers cultural practitioners and develops them as public health allies. Collaborative health promotion programs involving cultural experts can address barriers to participation and facilitate the creation of health behaviors in practical and meaningful ways.

This study provides a unique exploration of collaboration with cultural experts for health promotion program implementation and research. Findings must be considered in the context of their limitations. The small sample narrows the parameters of the findings. While the sample included both women and men, rural and urban areas, and a range of ages and experiences, it did not include certain demographics such as hula educators outside of Hawai‘i. Also, the focus of the Ola Hou program was cardiovascular health and specifically HTN management. It should not be assumed that hula educators would be as successful in other health promotion areas. It will be important in future research to understand if the hula educators’ success can be sustained through remote delivery, for example through internet-based hula training. It is also worthwhile to understand if there are specific health areas that the hula educators think might be applicable for the use of hula. Several respondents mentioned the benefits dancing the hula has for elders. This could have implications for diverse health promotion interventions such as fall prevention and reducing cognitive decline.

## CONCLUSION

Inroads to achieving health equity require finding new and better approaches to health promotion. For Indigenous and minority populations culturally-responsive programs are widely acknowledged as necessary and important. To meaningfully develop and implement these programs collaborations with, not only communities, but cultural experts are important. While alliance building and collaboration with cultural experts can be complex and time-consuming, they can deeply enrich health promotion programs. Findings from this study provides an important example of how collaborating with cultural experts can leverage cultural strengths, build health allies, and formulate unique research partnerships that can to create healthy behavioral change.

## Supplementary Material

daad022_suppl_Supplementary_Material
