# Supplementary material for: Developing culturally-responsive health promotion: insights from cultural experts
Source: Health Promot Int. 2023 Apr 17;38(2):daad022. doi: 10.1093/heapro/daad022 (PMC10108309; doi:10.1093/heapro/daad022)
Supplement: daad022_suppl_Supplementary_Material [file daad022_suppl_supplementary_material.docx]

Complete Hawaiian Culturally Responsive Educator Guidelines and Ola Hou Program Application

| Guideline Description | Guideline Example | Hula Educators application |
| --- | --- | --- |
| Incorporate cultural traditions, language, history, and values in meaningful holistic processes to nourish the emotional, physical, mental/intellectual, social, and spiritual well-being of the learning community that promote healthy *mauli* (life force) and *mana* (power, authority, priviledge) | Model culturally appropriate behavior in teaching | Establishing safe, familial learning environment and relationships between participants and between hula educator and participants |
| Maintain practices that perpetuate Hawaiian heritage, traditions, and language to nurture one’s mauli and perpetuate the success of the whole learning community | Provide opportunities to learn through observation and hands-on demonstration of cultural knowledge and skills | Learning of hula dances as means increasing cultural knowledge of Hawaiian language, history, and practices |
| Sustain respect for the integrity of one’s own cultural knowledge and provide meaningful opportunities to make new connections among other knowledge systems | Provide experiences that encourage learners to appreciate the uniqueness of other cultures | Sharing of heritage and various multi-cultural food during the *hoʻike* (traditional presentation of knowledge) at the completion of the class provides opportunity to appreciate different cultures |
| Instill a desire for lifelong exploration of learning, teaching, leading, and reflecting to pursue standards of quality and excellence | Demonstrate quality and excellence through product and performance | Inclusion of a traditional *ho‘ike* which included presentation of learned hula repertoire |
| Provide safe and supportive places to nurture the physical, mental/intellectual, social, emotional, and spiritual health of the total community | Utilize multiple instructional strategies and apply those strategies appropriately and flexibly in response to the instructional environment in which they are situated (i.e. singing, learning to speak Hawaiian) | Use of songs in the Hawaiian language and including singing of Hawaiian language songs in the warm-up walking-singing portion of classes |
| Foster understanding that culture and tradition, as constantly evolving systems, are grounded in the knowledge of the past to address the present and future | Make personal connections to cultural and traditional knowledge and to the application of that knowledge to validate teaching and learning styles | Use of place-based hula relevant to the participants as part of Ola Hou program dance repertoire |
| Engage in Hawaiian language opportunities to increase language proficiency and effective communication skills in a variety of contexts and learning situations | Engage learners in activities that increase language proficiency and confidence. (learning place names, native flora & fauna, singing Hawaiian songs) | Teaching place-based hula and including outings to historic areas. |
| Engage in activities independently or collaboratively with community members to perpetuate traditional ways of knowing, learning, teaching, and leading to sustain cultural knowledge and resources within the learning community | Provide opportunities for students to learn through observation and hands-on demonstrations of cultural knowledge and skills | Teaching place-based hula and including outings to historic areas. |
| Utilize multiple pathways and multiple formats to assess what has been learned and honor this process to nurture the quality of learning within the community | Utilize forms of assessment and evaluation other than written and standardize tests | Inclusion of ho‘ike presentation and blood pressure measurements in regular testing sessions |
| Support lifelong aloha for Hawaiian language, history, culture and values to perpetuate the unique cultural heritage of Hawai’i | Utilize locally relevant curriculum materials with which learners can readily identify, including materials prepared by Hawaiian authors | Utilization Hawaiian songs, subject matter and language to teach history, culture and values |
| Promote personal growth and development to strengthen cultural identity, academic knowledge and skills, *pono* (righteous, appropriate0 decision making, and ability to contribute to one’s self and family, and local and global communities. | Participate in, contribute to, and learn from local community events and activities in culturally appropriate ways. | Encourage awareness of local cultural and political activities related to “aloha ʻāina” love for the land. Present opportunities to share hula at community events such as assisted living facilities, health fairs, community fund-raisers |
| Develop an understanding of Hawaiian language, history, culture, and values through an indigenous perspective to foster a sense of self, place, community and global connection | Use local expertise, especially knowledgeable kūpuna (elders) as resource teachers/resources in classrooms and on excursions | Use of excursions to learn about songs used in dances. Use the knowledge of the participants to enrich discussions |
| Promote respect for how the Hawaiian cultural worldview contributes to diversity and global understanding to enhance onesʻs sense of self, family, and local and global communities | Integrate songs, dances, games, stories, and arts into learning | Naturally occuring with learning of new hula which includes songs, dances, stories, and cultural arts |
